# Effects of tumour necrosis factor and interleukin‐6 inhibitor therapy on exercise‐related visceral adipose tissue reduction in rheumatoid arthritis

**DOI:** 10.1113/EP093787

**Published:** 2026-06-16

**Authors:** Simon Jønck, Malte Lund Adamsen, Iben E. Rasmussen, Anna A. Lytzen, Mathilde Løk, Morten Asp Vonsild Lund, Benjamin Nerup, Lene Wohlfahrt Dreyer, Peter G. Jørgensen, Niels Vejlstrup, Lars Køber, Søren Jacobsen, Bente Klarlund Pedersen, Helga Ellingsgaard, Pil Højgaard, Ronan M. G. Berg, Regitse Højgaard Christensen

**Affiliations:** ^1^ Centre for Physical Activity Research Rigshospitalet, University of Copenhagen Copenhagen Denmark; ^2^ House of Research Holbæk Hospital Holbæk Denmark; ^3^ Copenhagen Center for Autoimmune and Connective Tissue Diseases (COPEACT) Rigshospitalet, University of Copenhagen Copenhagen Denmark; ^4^ Department of Cardiology Rigshospitalet, University of Copenhagen Copenhagen Denmark; ^5^ Department of Biomedical Sciences University of Copenhagen Copenhagen Denmark; ^6^ Center of Rheumatic Research Aalborg (CERRA), Department of Rheumatology Aalborg University Hospital Aalborg Denmark; ^7^ Department of Cardiology Herlev and Gentofte Hospital Herlev Denmark; ^8^ Department of Clinical Medicine, Faculty of Health and Medical Sciences University of Copenhagen Copenhagen Denmark; ^9^ Department of Internal Medicine M2, Rheumatology Holbæk Hospital Holbæk Denmark; ^10^ Center for Rheumatology and Spine Diseases Rigshospitalet Frederiksberg Denmark; ^11^ Department of Clinical Physiology and Nuclear Medicine Rigshospitalet, University of Copenhagen Copenhagen Denmark; ^12^ Neurovascular Research Laboratory, Faculty of Life Sciences and Education University of South Wales Pontypridd UK; ^13^ Department of Cardiology Zealand University Hospital Roskilde Denmark

**Keywords:** biologic disease‐modifying anti‐rheumatic drugs, glucose homeostasis, high‐intensity interval training, inflammation

## Abstract

Excess visceral adipose tissue (VAT) mass increases cardiovascular disease risk. Exercise supports overall health and VAT mass reduction in rheumatoid arthritis (RA). Unlike tumour necrosis factor (TNF), interleukin‐6 (IL‐6) is upregulated following exercise and is involved in free fatty acid mobilisation and might mediate the reduction in VAT mass. Given that treatment strategies for patients with RA include the inhibition of IL‐6 (IL‐6i) or TNF (TNFi), in this study we compared the effects of exercise on changes in VAT mass in patients with RA on stable IL‐6i or TNFi treatment. In this secondary analysis of a randomised controlled trial, 69 patients with RA on stable TNFi (*n* = 40) or IL‐6i (*n* = 29) therapy were stratified for treatment and assigned to a 12‐week supervised high‐intensity interval training (HIIT) intervention or no exercise (control). The primary objective was to investigate the effects of TNFi or IL‐6i therapy on exercise‐induced changes in VAT mass (in grams) from baseline to 12‐week follow‐up, assessed by abdominal MRI. Compared with the control group, HIIT did not change VAT mass in either TNFi {−34 g [95% confidence interval (CI), −206 to 139 g; *P* = 0.696]} or IL‐6i [−16 g [95% CI, −228 to 197 g; *P* = 0.882)] treatment groups. There was no interaction between IL‐6i and exercise on changes in VAT mass [18 g (95% CI, −256 to 292); interaction *P* = 0.895]. Exercise‐induced changes to the subcutaneous adipose tissue and insulin sensitivity were negligible and not significantly different between treatment modalities. In conclusion, a 12‐week HIIT intervention did not reduce VAT mass in patients with RA undergoing treatment with either TNFi or IL‐6i.

## Abstract (highlights)

- **What is the central question of this study?**
  Does a 12‐week supervised high‐intensity interval training intervention reduce visceral adipose tissue mass and improve glucose homeostasis in individuals with rheumatoid arthritis treated with biologic disease‐modifying anti‐rheumatic drugs?
- **What is the main finding and its importance?**
  Patients with rheumatoid arthritis on stable biologic disease‐modifying anti‐rheumatic drugs inhibiting tumour necrosis factor or interleukin‐6 activity seem to have an attenuated capability of exercise‐induced visceral adipose tissue mass loss. Likewise, insulin sensitivity was unaffected. Future research should explore alternative exercise modalities, longer interventions or additional metabolic factors that might contribute to visceral adipose tissue reduction in rheumatoid arthritis patients undergoing biological therapy.

## 1 INTRODUCTION

Rheumatoid arthritis (RA) is a chronic autoimmune disease linked via inflammatory activity to an increased cardiometabolic risk. Tumour necrosis factor (TNF) and interleukin‐6 (IL‐6) are pro‐inflammatory cytokines essential in driving the inflammation in RA (Smolen et al., 2016). Accordingly, TNF inhibitors (TNFi) and IL‐6 inhibitors (IL‐6i) are widely used biologic disease‐modifying anti‐rheumatic drugs (bDMARDs) in RA and are considered equal in terms of treatment effectiveness and safety (Lauper et al., 2020; Sepriano et al., 2020).

In addition to its well‐characterised pro‐inflammatory properties, IL‐6 is also known to exert several non‐inflammatory effects, including a modulatory role on visceral adipose tissue (VAT) mass. During exercise, skeletal muscles acutely secrete IL‐6 into the circulation independently of TNF (Steensberg et al., 2000, 2002), such that IL‐6 in blood increases exponentially in an intensity‐dependent manner (Berg, 2026; Fischer, 2006), which has been shown to mediate exercise‐induced VAT loss and promote free fatty acid mobilisation in humans (Trinh et al., 2021; Wedell‐Neergaard et al., 2019). On the contrary, TNF is suppressed by exercise training and is not thought to contribute to any exercise adaptations (Starkie et al., 2003). Yet, the role of exercise‐induced IL‐6 in adipose tissue regulation in patients with RA is unknown.

High‐intensity interval training (HIIT) maximises metabolic stress, which is thought to drive adaptations in adipose tissue (Maillard et al., 2018). HIIT has consistently been shown to reduce total and visceral fat mass in healthy and patient populations, with effects comparable to or greater than more time spent on moderate‐intensity regimens (Guo et al., 2023; Keating et al., 2017; Wewege et al., 2017), This occurs despite lower fat oxidation during the acute exercise bouts, probably reflecting the importance of cumulative energy expenditure and postexercise metabolic adaptations, rather than substrate utilisation during the exercise bout per se (Boutcher, 2011). However, given that excessive VAT mass is an independent predictor of cardiometabolic disease and disease activity in patients with RA (Giles et al., 2010), treatment with IL‐6i compared with TNFi might have different effects on adipose tissue regulation following HIIT.

In the present secondary analysis from a randomised controlled trial, we aimed to examine the effects of a 12‐week HIIT intervention on changes in VAT mass in patients with RA in stable IL‐6i or TNFi treatment. We previously showed that patients with RA in TNFi treatment exhibited significant cardiac adaptations to this intervention (Jønck et al., 2025). In contrast, this was not found in patients receiving IL‐6i treatment. Therefore, we hypothesised that relative to controls, concomitant treatment with IL‐6i compared with TNFi treatment would attenuate the exercise‐induced loss of VAT, measured by abdominal MRI.

## 2 MATERIALS AND Methods

### 2.1 Ethical approval

This study is based on a secondary analysis from the RABEX study (Jønck et al., 2023), which was approved by the Regional Scientific Ethical Committee (approval number H‐21010559, with amendments 86424, 87463 and 88044), the Danish Medicines Agency (Eudra‐CT 2021‐005287‐21) and registered on clinicaltrials.gov (NCT05215509). The study followed the principles outlined in the *Declaration of Helsinki*, and data were reported according to the CONSORT guidelines (Moher et al., 2012). All patients provided written informed consent prior to the baseline visit.

### 2.2 Participants

Individuals with RA aged between 18 and 69 years on stable (>4 months) treatment with TNFi or IL‐6i and a low disease activity score, defined as a disease activity score‐28 for RA with erythrocyte sedimentation rate (DAS‐28‐ESR) of ≤3.2 were eligible for participation. Furthermore, to minimise any residual effect, patients with corticosteroid use of >10 mg/day within 7 days and use of insulin or intramuscular corticosteroids within 3 weeks of enrolment were excluded. A complete list of inclusion and exclusion criteria has been published elsewhere (Jønck et al., 2023).

### 2.3 Design, setting and randomisation

The study was a single‐centre, open‐label, parallel‐group, assessor‐blinded randomised clinical trial. The study took place at the Centre for Physical Activity Research, Rigshospitalet, University of Copenhagen, Denmark, from January 2022 to August 2023. Subjects stratified for bDMARD treatment and sex were randomly allocated (1:1) in blocks of 10 to standard care with no exercise (control groups) or standard care with exercise (exercise groups). Following baseline assessments, patients were informed of group allocation by an unblinded study investigator.

### 2.4 Exercise intervention and standard care

The exercise groups underwent 3 sessions per week of 45 min of high‐intensity interval training (HIIT) on a cycle ergometer. All sessions were supervised by an experienced trainer. Patients wore a heart rate (HR) monitor watch during each session (Polar RS400; Polar, Kempele, Finland), and the watts were adjusted throughout the study according to the prespecified intensity. Each session consisted of a short warm‐up, followed by four rounds of 4 min at a target HR of ≥85% maximal HR, with 3 min of active rest between each interval. The session was concluded with an approximate 10 min cool‐down.

Standard care included routine visits at the RA outpatient clinic, to which each patient was affiliated before enrolment, independent of this study. An unblinded investigator contacted all patients at weeks 4 and 8 to record potential adverse events and compliance with treatment.

### 2.5 Experimental days

Two visits, the first at the Centre for Physical Activity Research and the second at the Department of Cardiology, Rigshospitalet, Copenhagen, Denmark, were conducted at baseline and repeated identically during follow‐up. The first visit, scheduled in the morning following an overnight fast of ≥10 h and abstention from strenuous exercise for 48 h, included a standard medical examination, blood samples, assessment of disease activity, questionnaires and an oral glucose tolerance test (OGTT). An abdominal MRI scan was performed at the second visit.

### 2.6 Medical examination, blood samples and disease activity

A medical history, examination and blood sampling were performed by a physician and followed standard procedures. Blood samples taken from the antecubital vein were used for measurements of high‐density lipoprotein, low‐density lipoprotein, total cholesterol, triglycerides, C‐peptide, glucose and insulin. All samples were analysed by standard procedures at the Department of Clinical Biochemistry, Rigshospitalet, Copenhagen, Denmark. Disease activity was assessed by DAS‐28‐ESR, the clinical disease activity index (CDAI) and visual analog scales (VAS) for pain, fatigue and global disease. DAS‐28‐ESR and CDAI provide a numerical score that combines swollen and tender joint count, patient global assessments along with the inflammatory marker ESR in the former and physician global assessments in the latter. A higher score indicates greater disease activity for both measurements (Arya et al., 2007). The VAS is a rating system (0–10 cm) to measure the impact of disease on given outcomes. A higher rating represents increased impact.

### 2.7 Questionnaires

Patient‐reported outcomes were assessed by self‐administered questionnaires including the Health Assessment Questionnaire with Disability Index (HAQ‐DI) and the RAND version of the Short Form 36 questionnaire (SF‐36) (Hays et al., 1993; Thorsen et al., 2001). The HAQ‐DI is composed of 20 questions in eight different domains, which assess disability and function, rating the level of difficulty from zero to three, where higher scores indicate more severe difficulty. The collected score is an average of the lowest rating from each domain. The SF‐36 is a 36‐item questionnaire covering eight topics of disease, from general health to fatigue and pain (Bjorner et al., 1998). Analysis was done by the RAND method, which standardises the different scales within the questionnaire to a scale ranging from 0 to 100 points, reverses negative scales such that scoring higher is always better, and averages across the domains. The questionnaire was aggregated into mental and physical components (Ware et al., 1994).

### 2.8 Oral glucose tolerance test

We performed a standardised 2‐h OGTT, in which participants orally ingested a fluid mixture containing 75 g of glucose and 293 mL of water within 2 min. Blood samples were analysed for C‐peptide, insulin and glucose levels before the ingestion and at time points 15, 30, 60, 90 and 120 min. Patients were placed in a bed in a supine or seated position during the test. The Matsuda index (Matsuda & DeFronzo, 1999), a measurement of whole‐body insulin sensitivity derived from evaluations of insulin and glucose levels during the OGTT, was used to assess insulin sensitivity (Wrench et al., 2024).

### 2.9 Magnetic resonance imaging

VAT and subcutaneous adipose tissue (SAT) imaging was carried out through a volumetric interpolated breath‐hold examination by a two‐point Dixon fat–water separation method, encompassing the entire thorax and abdomen in the axial plane. The midpoint of the T12/L1 and L5/S1 intervertebral discs was identified using RadiAnt Dicom Viewer 2023.1 (64‐bit) and all slices from T12/L1 to L5/S1 selected for analysis (Steele et al., 2021). SAT and VAT were evaluated by semi‐automatic calculations in sliceOmatic 5.0 Rev‐16c (TomoVision). If the unilateral dimensions of the patient exceeded the imaging boundaries of the scanner, the field of view was repositioned to include the full SAT area on one side of the body, but not the other. In this case, SAT was evaluated by quantifying the SAT area on one side of the body and multiplying it by two. To distinguish non‐adipose tissue from VAT, a histogram threshold was applied, with a lower limit set at 10. Subsequently, manual correction was performed by outlining the spinal column, skeletal muscle tissue, intramuscular fat and kidneys (Steele et al., 2021). In the event of a transition error between the upper and lower abdomen with missing overlap, correction involved averaging the values from 16 adjacent slices (8 above and 8 below) and multiplying by the number of missing frames. We adjusted the scans to ensure comparability using an equal number of slices from baseline and follow‐up from each patient. The same blinded investigator performed all analysis of MRI scans. To test for intraclass correlation, an abdominal MRI expert reanalysed a subset of 10 scans in a blinded manner. The total volume for SAT and VAT (in centimetres cubed) was converted to mass (in grams) by a multiplication factor of 0.920 g/mL (Richmond, 1985).

### 2.10 Physical activity and diet measurements

At baseline, patients were asked to fill out a self‐reported questionnaire regarding weekly structured exercise. Calorie intake was monitored by a self‐reported 3‐day dietary journal at the midway point of the study (week 6). Overall free‐living physical activity, including sedentary and moderate or vigorous activity, was measured using a 5‐day accelerometer placed on the right thigh and lower back (Axivity, AX3, Newcastle, UK) following the final visit.

### 2.11 Study outcomes

The primary outcome of this secondary analysis was the difference in change in VAT between the IL‐6i + exercise and the TNFi + exercise groups, relative to their control groups (interaction analysis). Secondary outcomes were analysed and interpreted in the same way and included SAT, the Matsuda index and area under the curve (AUC) plasma glucose, C‐peptide and insulin levels from the OGTT, and patient‐reported outcomes; HAQ‐DI and SF‐36. The analyses of the exploratory outcomes were identical to the primary and secondary outcomes and included lipid blood markers, CDAI score and VAS scores on pain, global disease and fatigue.

### 2.12 Statistical analysis

A prespecified sample size of 16 patients with RA in each group (*n* = 64 in total) was determined through a power calculation based on the primary outcome (Jønck et al., 2023). To account for anticipated dropouts and non‐adherence, we aimed to enrol 20 patients in each group (*n* = 80 in total). Given that the effect of IL‐6i versus TNFi on exercise‐induced changes in VAT was a secondary outcome, no power calculation was conducted for this specific measure. Statistical analysis was conducted in RStudio (R v.4.3.0; R Core Team, 2021). The continuous outcomes were analysed using a constrained baseline model for each bDMARD group (IL‐6i and TNFi) through a linear mixed model, using the lme4 package; this model natively handles repetitions of measurements on the same individual through a correlation matrix (Bates et al., 2015). To analyse the interaction effect, the linear model included group allocation (exercise or control), time (baseline or follow‐up) and treatment (TNFi or IL‐6i). The interaction effect refers to the difference in change between the groups IL‐6i + exercise and IL‐6i + control and between the groups TNFi + exercise and TNFi + control for a given outcome. Estimated marginal means and contrasts were calculated by the emmeans package (Lenth et al., 2023). Missing data were assumed to be missing at random. All patients who completed the baseline, regardless of adherence or follow‐up attendance, were incorporated in the intention‐to‐treat analysis. A per‐protocol analysis was performed, including only patients who adhered to the prespecified requirements of a minimum of 80% attendance to the intervention, a minimum of 80% bDMARD compliance, and completion of the follow‐up MRI. Statistical significance was set at the standard α‐level of *P *< 0.05. Baseline data are presented as means (±SD) and follow‐up data as estimated marginal means [95% confidence interval (CI)]. Given that this study analysed secondary outcomes, no multiplicity adjustments were made.

### 2.13 Patient and public involvement statement

Neither patients nor the public were involved in the design of this study.

## 3 RESULTS

The study enrolled 69 patients with RA, consisting of 40 in the TNFi treatment group and 29 in the IL‐6i treatment group. Eight patients were lost to follow‐up: five in the IL‐6i + exercise group, one in the IL‐6i + control group, and two in the TNFi + control group. Eight patients failed to meet the per‐protocol criteria. Specifically, four patients from the TNFi group and two from the IL‐6i group were unable to complete the exercise intervention successfully, and one patient each from the IL‐6i + control group and the TNFi + exercise group did not meet the bDMARD compliance criteria. Furthermore, two patients (one from each of the IL‐6i + exercise and IL‐6i + control groups) informed an unblinded investigator at the follow‐up visit that they had started using Wegovy^®^ (Novo Nordisk, Denmark), a weight loss medication not commercially available in Denmark at the onset of the study. These patients were also excluded from the per‐protocol analysis.

The demographics and clinical characteristics are shown in Table 1. Overall, the four groups appeared similar. The mean age was 53.5 (9.5) years, and 13 participants (19%) were men. The body mass index in the IL‐6i and TNFi groups was 27.5 (6.3) and 26.2 (5.2) kg/m^2^, respectively. Patients in the IL‐6i group had a seemingly higher HAQ‐DI score than those in the TNFi group, with scores of 0.8 (0.7) and 0.4 (0.4), respectively. Adherence to the exercise intervention was similar between the TNFi and IL‐6i groups, and compliance with bDMARD treatment was consistent across all four groups, while measurements of free‐living physical activity, total calorie intake and self‐reported weekly physical activity level at baseline did not vary significantly between groups (Jønck et al., 2025). Only patients (*n* = 9) in the IL‐6i group were receiving concomitant oral steroid treatment, with a mean dosage of 5 (2.0) mg. Four of those were on continuous treatment, whereas five patients had a treatment of prescription as needed.

**TABLE 1:** Baseline characteristics.

	IL‐6 inhibitor (*n* = 29)		TNF inhibitor (*n* = 40)	
Group	Exercise (*n* = 17)	No exercise (control) (*n* = 12)	Exercise (*n* = 20)	No exercise (control) (*n* = 20)
**Demographics**				
Female/male, *n*	14/3	10/2	16/4	16/4
Age, years	53.9 (7.1)	52.8 (10.4)	51.3 (10.9)	54.7 (9.5)
BMI, kg/m^2^	27.4 (6.7)	27.6 (5.7)	26.5 (5.2)	25.9 (5.2)
**RA characteristics**				
DAS28‐ESR	1.5 (1.1)	2.0 (0.9)	2.1 (0.7)	2.0 (0.9)
HAQ‐DI (0‐3)	0.6 (0.6)	1.1 (0.8)	0.4 (0.4)	0.4 (0.4)
CDAI	2.0 (2.1)	3.4 (2.5)	1.3 (1.3)	1.0 (1.4)
VAS pain, mm	22.9 (21.9)	36.6 (27.9)	20.8 (20.2)	17.4 (15.5)
VAS global disease, mm	31.3 (23.7)	43.5 (30.0)	23.9 (16.5)	19.5 (18.2)
VAS fatigue, mm	36.4 (30.9)	44.8 (29.2)	41.5 (24.9)	32.1 (25.7)
Duration of RA, years	14.6 (8.7)	13.3 (7.7)	15.0 (9.1)	18.6 (12.9)
Duration of bDMARD, years	7.5 [1.7, 11.5]	2.3 [1.5, 6.2]	4.6 [2.6, 7.1]	4.0 [2.2, 8.9]
**Metabolic measurements**				
VAT, g	2128 (1655)	2105 (1189)	2093 (1554)	1978 (1359)
SAT, g	3559 (1645)	3136 (1509)	3327 (1379)	3150 (1456)
OGTT, glucose AUC, mmol min/L	806.9 (176.9)	828.3 (328.5)	815.2 (177.5)	896.9 (362.3)
OGTT, insulin AUC, nmol min/L	60.9 (41.3)	60.5 (62.4)	50.8 (34.7)	39.9 (22.7)
OGTT, c‐peptide AUC, nmol min/L	313.3 (113.5)	321.5 (159.1)	288.3 (126.1)	254.9 (80.5)
Matsuda index	4.9 (2.5)	4.1 (2.1)	6.5 (3.6)	5.8 (3.5)
Total cholesterol, mmol/L	5.5 (0.9)	4.8 (1.2)	4.6 (0.8)	5.2 (0.9)
HDL, mmol/L	1.6 (0.5)	1.5 (0.49	1.7 (0.5)	1.8 (0.4)
LDL, mmol/L	3.4 (0.8)	2.8 (0.9)	2.6 (0.6)	3.0 (0.7)
Triglycerides, mmol/L	1.4 (0.6)	1.3 (0.8)	0.9 (0.5)	1.0 (0.7)
**General health**				
SF‐36_physical_	40.5 (9.7)	38.0 (10.2)	47.7 (7.6)	47.8 (7.1)
SF‐36_mental_	55.14 (4.6)	47.1 (12.4)	47.3 (13.1)	51.8 (8.1)
**Concomitant medications**				
Oral steroid, *n* (%)	4 (23.5)	5 (41.7)	0 (0)	0 (0)
csDMARD, *n* (%)	5 (29.4)	3 (25.0)	14 (70.0)	14 (70.0)
NSAID, *n* (%)	13 (76.5)	8 (66.6)	8 (40.0)	5 (25.0)

*Note*: Values are depicted as the mean (SD), median [quartile 1, quartile 3] or number (percentage).

Abbreviations: AUC, area under the curve; bDMARD, biologic disease‐modifying anti‐rheumatic drug; BMI, body mass index; CDAI, clinical disease activity index; csDMARD, conventional synthetic disease‐modifying anti‐rheumatic drug; DAS28‐ESR, Disease Activity Score‐28 for rheumatoid arthritis with erythrocyte sedimentation rate; HAQ‐DI, health assessment questionnaire disability index; HDL, high‐density lipoprotein; IL‐6, interleukin‐6; LDL, low‐density lipoprotein; NSAID, non‐steroidal anti‐inflammatory drug; OGTT, oral glucose tolerance test; RA, rheumatoid arthritis; SAT, subcutaneous adipose tissue; SF‐36, Short Form 36 questionnaire; TNF, tumour necrosis factor; VAS, visual analog scale; VAT, visceral adipose tissue.

### 3.1 Visceral and subcutaneous adipose tissue mass

**FIGURE 1:**
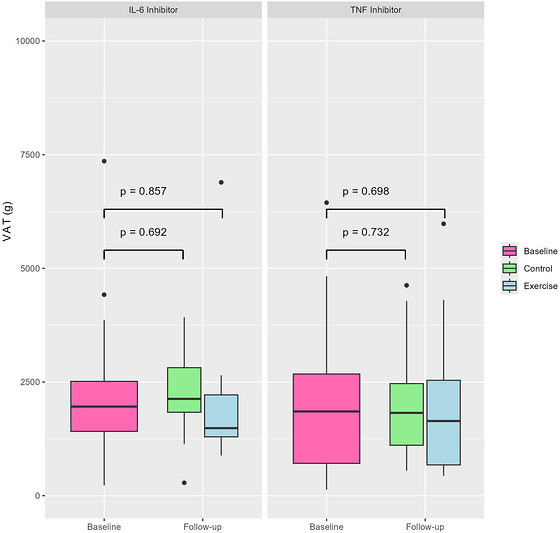
The estimated marginal means change in visceral adipose tissue (VAT) mass from baseline to 12‐weekfollow‐up in the intention‐to‐treat population. Black circles indicate individual outliers. Abbreviations: IL‐6i, interleukin‐6 inhibitor; TNFi, tumour necrosis factor inhibitor.

**FIGURE A1:**
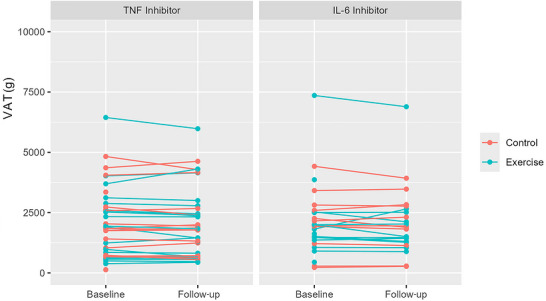
Visceral adipose tissue (VAT) at baseline and follow‐up for each group, coloured for intervention. Measurements from the same participant are connected by a line. Abbreviations: IL‐6i, interleukin‐6 inhibitor; TNFI, tumour necrosis factor inhibitor.

There was no significant difference in the change in VAT mass between the exercise and control groups for patients on TNFi [−34 g (95% CI, −206 to 139; *P* = 0.696)] or patients on IL‐6i [−16 g (95% CI, −228 to 197; *P* = 0.882); Table 2; Figure 1; Appendix Figure A1]. We found no modifying effect of IL‐6i treatment on exercised‐induced changes to VAT mass (interaction *P* = 0.895; Table 2). We found similar within‐group changes in VAT mass from baseline to follow‐up, with no significant differences detected across all four groups (Appendix Table A1).

**TABLE 2:** Between group difference in mean change and interaction between IL‐6 inhibitor and exercise.

	IL‐6 inhibitor (*n* = 29)				TNF inhibitor (*n* = 40)				
	Baseline	Follow‐up		Difference in mean change; exercise vs. control [95% CI] (*P*‐value)	Baseline	Follow‐up		Difference in mean change; exercise vs. control [95% CI] (*P*‐value)	
Group	Pooled	No exercise (control) (*n* = 12)	Exercise (*n* = 17)		Pooled	No exercise (control) (*n* = 20)	Exercise (*n* = 20)		Interaction^a^ [95% CI] (*P*‐value)
**Primary outcome**									
VAT, g	2118 (1440)	2239 (1077)	2093 (1611)	−16 [−228 to 197] (0.882)	2034 (1439)	2042 (1312)	2001 (1502)	−34 [−206 to 139] (0.696)	18 [−256 to 292] (0.895)
**Secondary outcomes**									
SAT, g	3380 (1572)	3664 (2222)	3814 (1814)	93 [−285 to 471] (0.62)	3234 (1404)	3071 (1169)	3171 (1331)	3 [−296 to 301] (0.98)	90 [−391 to 572] (0.70)
Matsuda index	4.6 (2.4)	4.1 (2.1)	5.3 (3.4)	0.6 [−1.1 to 2.3] (0.494)	6.2 (3.5)	6.6 (4.2)	5.9 (2.5)	−1.0 [−2.4 to 0.4] (0.151)	1.60 [−0.6 to 3.8] (0.149)
OGTT Glucose AUC, (mmol min)/L	815.8 (245.7)	831.2 (354.8)	772.7 (156.9)	−31.1 [−174.5 to 112.4] (0.666)	856.1 (284.6)	864.4 (299.2)	799.4 (125.3)	7.7 [−105.5 to 120.9] (0.892)	−38.8 [−221.5 to 144.0] (0.672)
OGTT Insulin AUC, (nmol min)/L	60.8 (50.0)	70.3 (57.2)	50.2 (33.5)	−22.9 [−44.2 to −1.6] (0.036)	45.4 (29.4)	36.4 (20.7)	50.1 (28.3)	1.6 [−15.2 to 18.4] (0.850)	−24.5 [−51.6 to 2.7] (0.076)
OGTT C‐peptide AUC, (nmol min)/L	316.7 (131.7)	354.8 (161.3)	320.5 (132.6)	−30.2 [−80.4 to 19.9] (0.232)	271.6 (105.8)	25.4 (88.2)	290.5 (97.7)	−4.1 [−43.6 to 35.3] (0.834)	−26.1 [−89.9 to 37.7] (0.416)
HAQ‐DI	0.8 (0.7)	0.9 (0.7)	0.5 (0.5)	−0.2 [−0.3 to −0.03] (0.014)	0.4 (0.4)	0.4 (0.4)	0.4 (0.5)	−0.02 [−0.1 to 0.1] (0.714)	−0.1 [−0.3 to 0.02] (0.083)
SF‐36_physical_	39.5 (9.8)	40.3 (9.7)	44.7 (8.8)	3.4 [−1.8 to 8.7] (0.19)	47.8 (7.3)	45.96 (8.6)	46.4 (8.8)	1.0 [−3.3 to 5.2] (0.640)	2.4 [−4.3 to 9.2] (0.47)
SF‐36_mental_	51.8 (9.4)	45.1 (11.5)	54.3 (9.7)	0.9 [−7.0 to 8.7] (0.828)	49.7 (10.8)	53.8 (8.8)	50.6 (9.9)	1.5 [−4.8 to 7.9] (0.632)	−0.6 [−4.3 to 3.9] (0.893)
**Exploratory outcomes**									
CDAI	2.6 (2.3)	4.8 (5.9)	1.7 (1.4)	−1.8 [−4.4 to 0.8] (0.171)	1.2 (1.3)	2.1 (3.4)	1.24 (1.2)	−1.1 [−3.3 to 1.09] (0.299)	−0.7 [−4.0 to 2.7] (0.689)
VAS pain	28.6 (25.1)	33.3 (30.6)	17.6 (13.4)	−2.7 [−14.8 to 9.4] (0.658)	19.0 (17.7)	20.2 (15.5)	20.7 (20.9)	−4.1 [−13.0 to 5.7] (0.403)	1.4 [−14.2 to 17.0] (0.854)
VAS global disease	36.3 (26.7)	42.4 (28.3)	28.9 (19.2)	−0.1 [−12.1 to 11.9] (0.992)	21.6 (17.4)	23.1 (19.9)	25.8 (22.1)	−3.1 [−12.2 to 6.62] (0.523)	3.0 [−12.4 to 18.6] (0.693)
VAS fatigue	39.9 (29.9)	50.0 (33.1)	37.2 (27.5)	0.52 [−14.1 to 15.2] (0.943)	36.5 (25.4)	41.5 (28.3)	44.3 (31.0)	−6.1 [−17.9 to 5.8] (0.310)	6.6 [−12.3 to 25.4] (0.487)
Total cholesterol, mmol/L	5.2 (1.0)	4.7 (0.7)	5.2 (0.7)	0.03 [−0.5 to 0.5] (0.914)	4.9 (0.9)	4.9 (1.2)	4.7 (0.9)	0.2 [−0.1 to 0.6] (0.222)	−0.2 [−0.8 to 0.4] (0.500)
HDL, mmol/L	1.6 (0.4)	1.5 (0.3)	1.6 (0.4)	0.1 [−0.3 to 0.4] (0.649)	1.8 (0.4)	1.9 (0.5)	1.8 (0.4)	−0.1 [−0.3 to 0.29] (0.601)	0.1 [−0.3 to 0.6] (0.496)
LDL, mmol/L	2.8 (0.7)	2.9 (0.8)	2.7 (0.8)	−0.1 [−0.5 to 0.3] (0.527)	3.2 (0.9)	2.8 (0.6)	3.3 (0.7)	0.2 [−0.1 to 0.5] (0.287)	−0.3 [−0.8 to 0.2] (0.250)
Triglyceride mmol/L	1.4 (0.7)	1.2 (0.6)	1.1 (0.4)	−0.1 [−0.5 to 0.3] (0.494)	0.9 (0.6)	0.9 (0.4)	0.9 (0.4)	0.1 [−0.2 to 0.3] (0.701)	0.01 [−0.7 to 0.3] (0.439)

*Note*: Values are presented as estimated marginal means with (SD) or [95% CI] in the intention‐to‐treat population.

Abbreviations: AUC, area under the curve; CDAI, clinical disease activity index; HAQ‐DI, health assessment questionnaire disability index; HDL, high‐density lipoprotein; IL‐6, interleukin‐6; LDL, low‐density lipoprotein; OGTT, oral glucose tolerance test; SAT, subcutaneous adipose tissue; SF‐36, Short Form 36 questionnaire; TNF, tumour necrosis factor; VAS, visual analog scale; VAT, visceral adipose tissue.

^a^Interaction of IL‐6i and exercise across all four groups.

The differences in SAT changes from baseline to follow‐up between the exercise and control groups were negligible for both treatment modalities [TNFi, 3 g (95% CI, −293 to 299; *P* = 0.985); and IL‐6i, 82 g (95%CI, −282 to 446; *P* = 0.654); Table 2]. The intraclass correlation was excellent for both VAT [0.99 (95% CI, 0.98 to 0.99)] and SAT [0.98 (95% CI, 0.98 to 0.99)].

### 3.2 Oral glucose tolerance test

Compared with the control groups, the Matsuda index did not change with exercise across both bDMARDs [TNFi, −1.0 (95% CI, −2.4 to 0.4; *P* = 0.151); and IL‐6i, 0.6 (95% CI, −1.1 to 2.2: *P* = 0.494); Table 2], and no within‐group changes were observed (Appendix Table A1). The AUC values for glucose, insulin and C‐peptide were unaffected by exercise across bDMARDs (Table 2).

### 3.3 Patient‐reported outcomes

In the TNFi group, HAQ‐DI was not significantly affected by exercise compared with the control group, with a between‐group difference of −0.02 (95% CI, −0.1 to 0.1; *P* = 0.714). In contrast, exercise significantly improved HAQ‐DI in the IL‐6i group compared with the control group [−0.2 (95% CI, −0.3 to −0.03; *P* = 0.014); Table 2]. Only the IL‐6i + exercise group showed a significant within‐group improvement in HAQ‐DI from baseline to follow‐up (Appendix Table A1). The SF‐36_physical_ component scores did not change significantly between the exercise and control groups for both bDMARDs [TNFi, 1.0 (95% CI, −3.3 to 5.2; *P* = 0.643); and IL‐6i, 3.4 (95% CI, −1.8 to 8.7; *P* = 0.198)]. Similar insignificant results were observed for the SF‐36_mental_ component (Table 2).

### 3.4 Other exploratory outcomes

We found no significant changes in the CDAI score, VAS scores or blood lipids between the exercise and control groups across both TNFi and IL‐6i treatments (Table 2). The interaction analyses of IL‐6i and exercise were insignificant for all exploratory outcomes (Table 2).

The per‐protocol analysis on within‐group changes from baseline to 12‐week follow‐up aligned with the intention‐to‐treat analysis (Appendix Tables A1 and A2).

## 4 Discussion

This secondary analysis from a randomised controlled trial examined the effects of concomitant IL‐6i or TNFi treatment on exercised‐induced loss of VAT mass. We did not find any effect of exercise on changes in VAT regardless of whether patients were treated with IL‐6i or TNFi.

The overall lack of an effect of exercise on VAT in this study is surprising because it is well established that high‐intensity aerobic exercise, similar to the intervention in this study, targets VAT loss in patients both with and without RA (Ismail et al., 2012; Vissers et al., 2013; Yekini & Grace, 2023). We previously reported that cardiac adaptation and changes in maximal O_2_ uptake in response to exercise training differed among the groups in the same trial (Jønck et al., 2025), further emphasising the unexpected nature of these results. However, less is known about effects of exercise on VAT in patients with RA concomitantly treated with bDMARDs, Notably, in a non‐RA population, it has been shown that IL‐6i attenuated exercise‐induced VAT reduction following a 12‐week HIIT programme compared with placebo (Wedell‐Neergaard et al., 2019). Subsequently, it has been found that IL‐6i affects exercise‐induced fatty acid mobilisation (Trinh et al., 2021), which is thus consistent with the observed lack of an effect on VAT in patients receiving IL‐6i observed here.

Despite some studies have found that year‐long TNFi treatment over longer periods increases adipose tissue mass compared with conventional synthetic DMARDs (Engvall et al., 2010), the lack of exercise‐induced VAT loss in the TNFi group was still unexpected, given that TNF is not known to mediate any lipolytic effects of exercise (Starkie et al., 2003). Previous studies on RA patients not treated with bDMARDs have demonstrated notable changes in body composition following exercise interventions (Azeez et al., 2020; Joo et al., 2022; Morsley et al., 2018), suggesting that the present findings might reflect the unique characteristics of this patient cohort. This is underlined by dual‐energy X‐ray absorptiometry results from this trial showing no improvements in total fat mass or total lean mass following exercise across bDMARD treatments (Jønck et al., 2025). Given that patients with RA who require bDMARD treatment typically suffer from an unacceptable disease activity, thus potentially representing a selected group with a higher degree of rheumatoid cachexia than other RA patients, and increased disease activity is associated with less physical activity (Hernández‐Hernández et al., 2014), the absence of an exercise effect on VAT mass in this study might reflect an insufficient ability to perform exercise training in these patients. Given that the exercise‐induced IL‐6 from skeletal muscles depends on both the total muscle mass involved and the intensity and duration of exercise (Berg, 2026; Fischer, 2006), this could negatively impact the ability of the skeletal muscle to mount a sufficient IL‐6 release to elicit any effects on VAT. Nevertheless, adherence to the exercise intervention was high across both the IL‐6i and TNFi groups, with a median time of 13.9 and 13.4 min of ≥85% maximal HR per session, suggesting that the observed lack of effect of exercise on VAT is more likely to be attributable to an unknown influence of bDMARD treatment itself on the adaptive response to exercise. At present, the mechanisms through which bDMARDs might attenuate exercise‐induced changes in VAT remain unclear. Given that this study included few patients with VAT of >5000 g, the generalisability to obese patients is limited.

It is well established that glycaemic control as measured by the Matsuda index reflects an important component of the metabolic benefits of exercise training (Mourier et al., 1997). However, although 28 participants had a baseline Matsuda index of <4.3, suggestive of mild insulin resistance (Takahara et al., 2013), no significant improvement in whole‐body insulin sensitivity was observed in either the TNFi or the IL‐6i treatment group. These results align with other studies that combined exercise and IL‐6i in individuals without RA, suggesting that long‐term glucose metabolism remains unaffected by exercise‐induced IL‐6 (Ellingsgaard et al., 2020; Wedell‐Neergaard et al., 2019). This contrasts with the acute effects of increased glucose disposal during recombinant human IL‐6 infusions mimicking the plasma concentrations of strenuous exercise (Carey et al., 2006; Febbraio et al., 2004). Nevertheless, the Matsuda Index was higher, in the TNFi than in the IL‐6i treatment group at baseline, which might indeed reflect an insulin‐sensitising effect of TNFi, given the well‐known effect of TNF on skeletal muscle insulin sensitivity (Plomgaard et al., 2005), but potential confounders, such as between‐group differences in disease severity or duration, might also have contributed. Of note, the change in VAT did not depend on the Matsuda index in an ancillary analysis (data not shown).

In the patient‐reported outcomes, exercise significantly improved HAQ‐DI compared with control in the IL‐6i, but not in TNFi patients. However, the IL‐6i and exercise interaction effect was insignificant. The effect of exercise on HAQ‐DI in RA patients shows mixed results. Compared with the controls, the change in HAQ‐DI of 0.2 following exercise in IL‐6i patients is in line with previous findings in supervised RA exercise trials ranging from 4 to 16 weeks (Baillet et al., 2009; Flint‐Wagner et al., 2009). Conversely, a supervised 20‐week exercise study found no effect of exercise on HAQ‐DI (Lange et al., 2019). The discrepancy of HAQ‐DI changes between TNFi and IL‐6i patients following exercise might be explained by a somewhat higher baseline score for the IL‐6i patients.

The HIIT intervention did not affect blood lipids. However, the relatively normal baseline blood lipid levels in this cohort might have limited the potential for further improvement, although exercise training has previously been shown to improve lipid profiles even in normolipidaemic populations. Thus, the absence of an effect in the present study might possibly also reflect disease‐specific factors or the influence of concomitant biologic therapy (Kodama et al., 2007).

The study has several limitations. First, given that change in VAT was a secondary outcome, we did not enrol patients with RA based on an abdominal obesity criterion. Second, given that patients were stratified for bDMARD treatment prior to randomisation, inherent differences between patients in the TNFi and IL‐6i treatment groups pose a risk of residual confounding. The comparability between bDMARDS remains limited owing to differences in disease severity as expressed by HAQ, VAS global and CDAI. Randomising to bDMARD treatment was not feasible, and the design was chosen to ensure that participants adhered to both the bDMARD treatment and the intervention. Third, blinding of the exercise intervention was not possible, which could have affected patient‐reported outcomes, although unlikely to be different across bDMARDs. Nonetheless, no differences in the patient‐reported outcomes were found. Fourth, given that this was a secondary analysis, we did not incorporate a power analysis based on changes in VAT. This might have caused the study to be underpowered, and is likely to have increased the risk of a type II error (Berg et al., 2024). Furthermore, the assigned target of a minimum of 16 patients in each group was not met. Fifth, we did not include the habitual exercise level in the eligibility criteria. Although the intervention was designed to provide a substantial metabolic stimulus, participants already engaged in regular strenuous physical activity might have had limited capacity for further VAT reduction, potentially attenuating the observed effects. Sixth, the randomisation of the patients on concomitant IL‐6i treatment was skewed. This is likely to have occurred because the fixed block size (*n* = 10) was too large given the overall size of the study and the stratification by sex prior to randomisation. Finally, patient and public involvement was not included in the planning of this study.

## 5 CONCLUSION

In conclusion, a 12‐week HIIT exercise intervention did not elicit a change in VAT mass in patients with RA in concomitant treatment with either TNFi or IL‐6i, and we found no modifying interaction effect of IL‐6i and exercise.

## AUTHOR CONTRIBUTIONS

Simon Jønck, Malte Lund Adamsen, Ronan M. G. Berg, Bente Klarlund, Helga Ellingsgaard and Regitse Højgaard Christensen designed the study. Simon Jønck, Malte Lund Adamsen, Iben E. Rasmussen, Anna A. Lytzen and Mathilde Løk collected the data. All authors interpreted the data. Simon Jønck and Malte Lund Adamsen performed the analyses. Simon Jønck, Malte Lund Adamsen and Regitse Højgaard Christensen wrote the first draft. All authors revised and approved the final version of the manuscript and agree to be accountable for all aspects of the work in ensuring that questions related to the accuracy or integrity of any part of the work are appropriately investigated and resolved. All persons designated as authors qualify for authorship, and all those who qualify for authorship are listed.

## CONFLICT OF INTEREST

Malte Lund Adamsen has received a speaking fee from Novartis. Lene Wohlfahrt Dreyer has received research grant/research support (to the institution) from BMS and AbbVie, and speaker's bureau from Eli Lilly and Galderma. No other authors have any conflicts of interest to declare.

## Data Availability

The data underlying this article will be shared on reasonable request to the corresponding author.

## Appendix

**TABLE A1:** Intention‐to‐treat population.

	IL‐6 inhibitor (*n* = 29)		TNF inhibitor (*n* = 40)	
Group	No exercise (control) (*n* = 12)	Exercise (*n* = 17)	No exercise (control) (*n* = 20)	Exercise (*n* = 20)
∆VAT, g	−63 (−225 to 99)	−72 (−220 to 77)	−22 (−150 to 106)	−56 (−174 to 62)
∆SAT, g	−76 (−362 to 209)	17 (−231 to 264)	−83 (−298 to 131)	−81 (−289 to 127)
∆Matsuda index	0.2 (−1.1 to 1.4)	0.7 (−0.4 to 1.9)	0.2 (‐0.9 to 1.3)	−0.8 (−1.8 to 0.1)
∆OGTT Glucose AUC, (mmol min)/L	2.9 (−105.1 to 110.9)	−28.2 (−122.5 to 66.2)	−23.5 (−106.5 to 59.4)	−15.8 (−92.9 to 61.3)
∆OGTT Insulin AUC, (nmol min)/L	6.8 (−9.3 to 22.8)	−16.1 (−30.1 to 2.2)	−2.3 (−14.6 to 10.1)	−0.7 (−12.2 to 10.8)
∆OGTT C‐peptide AUC, (nmol min)/L	22.2 (−15.6 to 59.9)	−8.0 (−41.1 to 24.9)	6.4 (−22.6 to 35.3)	2.3 (−24.6 to 29.1)
∆HAQ‐DI	−0.03 (−0.1 to 0.03)	−0.2 (−0.3 to −0.1)	0 (−0.1 to 0.1)	−0.02 (−0.1 to 0.1)
∆SF‐36_physical_	1.9 (−2.0 to 5.9)	5.3 (1.9 to 8.8)	−2.3 (−5.4 to 0.7)	−1.3 (−4.3 to 1.7)
∆SF‐36_mental_	−1.7 (−7.6 to 4.1)	−0.9 (−6.0 to 4.2)	1.8 (−2.8 to 6.3)	3.3 (−1.2 to 7.8)
∆CDAI	1.4 (−0.5 to 3.4)	−0.4 (−2.1 to 1.3)	1.0 (−0.5 to 2.5)	−0.1 (−1.6 to 1.4)
∆VAS pain	−4.3 (−13.4 to 4.9)	−6.9 (−14.9 to 1.0)	4.1 (−2.9 to 11.1)	−0.1 (−6.9 to 6.8)
∆VAS global disease	−3.2 (−12.3 to 5.8)	−3.3 (−11.2 to 4.6)	5.0 (−1.9 to 11.9)	1.9 (−4.9 to 8.7)
∆VAS fatigue	1.3 (−9.7 to 12.3)	1.8 (−7.8 to 11.5)	8.9 (0.4 to 17.4)	2.8 (−5.5 to 11.1)
∆Total cholesterol, mmol/L	−0.2 (−0.5 to 0.2)	−0.2 (−0.5 to 0.2)	−0.1 (−0.4 to 0.2)	0.1 (−0.2 to 0.4)
∆HDL, mmol/L	−0.04 (−0.3 to 0.2)	0.04 (−0.2 to 0.3)	0.1 (−0.1 to 0.3)	0.1 (−0.1 to 0.2)
∆LDL, mmol/L	0 (−0.3 to 0.3)	−0.1 (−0.4 to 0.1)	−0.1 (−0.3 to 0.2)	0.1 (−0.1 to 0.3)
∆Triglycerides, mmol/L	−0.2 (−0.4 to 0.1)	−0.3 (−0.5 to −0.1)	−0.03 (−0.2 to 0.3)	0.02 (−0.2 to 0.2)

*Note*: Within‐group mean changes from baseline to 12‐week follow‐up in the intention‐to‐treat population. Values represent the mean and 95% confidence interval.

Abbreviations: AUC, area under the curve; CDAI, clinical disease activity index; HAQ‐DI, health assessment questionnaire disability index; HDL, high‐density lipoprotein; IL‐6, interleukin‐6; LDL, low‐density lipoprotein; OGTT, oral glucose tolerance test; SAT, subcutaneous adipose tissue; SF‐36, Short Form 36 questionnaire; TNF, tumour necrosis factor; VAS, visual analog scale; VAT, visceral adipose tissue.

**TABLE A2:** Per‐protocol population.

	IL‐6 inhibitor (*n* = 29)		TNF inhibitor (*n* = 40)	
Group	No exercise (control) (*n* = 9)	Exercise (*n* = 9)	No exercise (control) (*n* = 17)	Exercise (*n* = 15)
∆VAT, g	−51 (−263 to 120)	−41 (−222 to 140)	−11 (−151 to 129)	−84 (−225 to 56)
∆SAT, g	−62 (−397 to 273)	62 (−234 to 358)	−26 (−255 to 203)	−85 (−331 to 161)
∆Matsuda index	0.6 (−0.8 to 1.9)	0.9 (−0.34 to 2.3)	0.1 (−0.9 to 1.2)	−0.5 (−1.5 to 0.5)
∆OGTT Glucose AUC, (mmol min)/L	41.4 (−64.3 to 146.9)	−32.1 (−132.1 to 67.9)	−24.2 (−98.9 to 50.7)	−14.5 (−92.8 to 62.0)
∆OGTT Insulin AUC, (nmol min)/L	9.1 (−6.9 to 24.9)	−14.4 (−29.4 to 0.7)	−1.9 (−13.2 to 9.4)	−0.1 (−11.8 to 11.6)
∆OGTT C‐peptide AUC, (nmol min)/L	7.9 (−32.5 to 48.3)	−15.7 (−53.9 to 22.6)	8.6 (−20.1 to 37.1)	−3.9 (−33.6 to 25.7))
∆HAQ‐DI	−0.02 (−0.1 to 0.1)	−0.1 (−0.2 to −0.1)	0.01 (−0.1 to 0.1)	−0.03 (−0.1 to 0.1)
∆SF‐36_physical_	1.9 (−2.6 to 6.5)	5.4 (1.0 to 9.6)	−2.7 (−5.9 to 0.)	−0.5 (−4.1 to 3.1)
∆SF‐36_mental_	0.1 (−6.4 to 6.6)	−2.1 (−8.2 to 4.1)	2. (−2.6 to 6.6)	4.9 (−0.2 to 10.1)
∆CDAI	−0. (−2.70 to 1.4)	0.1 (−1.8 to 2.1)	1.0 (−0.5 to 2.5)	−0.5 (−2.2 to 1.1)
∆VAS pain	−6.3 (−15.1 to 2.5)	−3.8 (−12.1 to 4.6)	4.4 (−1.9 to 10.6)	0.4 (−6.6 to 7.3)
∆VAS global disease	−6.4 (−14.2 to 1.5)	−0.1 (−7.6 to 7.3)	3.5 (−2.1 to 9.1)	−0.4 (−6.6 to 5.8)
∆VAS fatigue	0.8 (−11.1 to 12.7)	−2.2 (−13.5 to 9.1)	9.9 (1.4 to 18.3)	1.3 (−8.1 to 10.7)
∆Total cholesterol, mmol/L	−0.2 (−0.6 to 0.2)	−0.1 (−0.5 to 0.3)	−0.1 (−0.4 to 0.2)	0.2 (−0.1 to 0.5)
∆HDL, mmol/L	−0.1 (−0.4 to 0.3)	0.1 (−0.3 to 0.4)	0.1 (−0.1 to 0.3)	0.1 (−0.2 to 0.3)
∆LDL, mmol/L	0.02 (−0.3 to 0.4)	−0.1 (−0.4 to 0.3)	−0.1 (−0.3 to 0.2)	0.1 (−0.1 to 0.4)
∆Triglycerides, mmol/L	−0.2 (−0.4 to 0.1)	−0.3 (−0.6 to −0.1)	−0.02 (−0.2 to 0.2)	0.04 (−0.2 to 0.3)

*Note*: Within‐group mean changes from baseline to week 12 in the per‐protocol population. Values are depicted as the mean (95% confidence interval).

Abbreviations: AUC, area under the curve; CDAI, clinical disease activity index; HAQ‐DI, health assessment questionnaire disability index; HDL, high‐density lipoprotein; IL‐6, interleukin‐6; LDL, low‐density lipoprotein; OGTT, oral glucose tolerance test; SAT, subcutaneous adipose tissue; SF‐36, Short Form 36 questionnaire; TNF, tumour necrosis factor; VAS, visual analog scale; VAT, visceral adipose tissue.
